# MicroRNA miR-497 is closely associated with poor prognosis in patients with cerebral ischemic stroke

**DOI:** 10.1080/21655979.2021.1940073

**Published:** 2021-06-21

**Authors:** Changyang Zhong, Congguo Yin, Guozhong Niu, Li Ning, Jinbo Pan

**Affiliations:** a Department of Neurology, Hangzhou Third People’s Hospital, Hangzhou, Zhejiang, China; b Department of Neurology, Hangzhou First People’s Hospital, Hangzhou, Zhejiang, China; c Nursing Department, Hangzhou First People’s Hospital, Hangzhou, Zhejiang, China; d Department of ICU, Hangzhou Third People’s Hospital, Hangzhou, Zhejiang, China

**Keywords:** Ischemic stroke, miR-497, oxidative stress response, neuronal apoptosis

## Abstract

Cerebral ischemic stroke (CIS) is the most common type of stroke, which is highly hazardous. This investigation aims to analyze the correlation of miR-497 with CIS, so as to provide reliable evidence for clinical response to CIS and lay a solid foundation for follow-up research. Eighty-nine CIS patients and 39 concurrent physical examinees selected between June 2017 and October 2018 were enrolled as the research participants. Additionally, SD rats with increased miR-497 expression and normal SD rats were purchased for CIS modeling to observe the clinical implications of miR-497 in CIS, as well as the water content of brain tissue and neuronal apoptosis of rats. miR-497 expression was lower in CIS patients than in physical examinees, and that in patients with complete stroke (CS) was the lowest, which increased after treatment. As determined by the receiver operating characteristic curve (ROC) analysis, miR-497 had an outstanding diagnostic efficacy for CIS and was negatively correlated with the National Institutes of Health Stroke Scale (NIHSS) and MDA concentration, while positively related to SOD concentration. Prognostic follow-up demonstrated that decreased miR-497 expression in patients after treatment predicted an increased risk of prognostic death and recurrence. However, observed in rats, the water content of the brain tissue of rats with increased miR-497 expression was reduced, and the neuronal apoptosis rate of the brain tissue was inhibited. Taken together, with low expression in CIS, miR-497 is strongly related to CIS progression and is a candidate CIS marker.

## Graphical abstract

**Figure:**
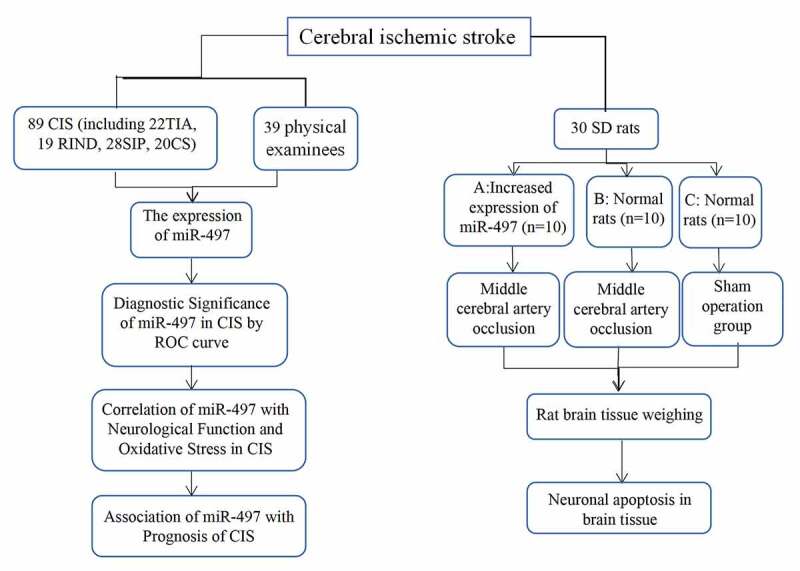

## 1. Introduction

Stroke, as an acute cerebrovascular disease with high incidence among middle-aged and elderly people in the world, poses a great threat to the life and health of patients [1]. The pathogenesis of stroke has been clinically recognized as the failure of blood flow to complete normal cerebral circulation due to cerebral vascular rupture or obstruction, resulting in oxidative stress and inflammatory damage to the brain tissue [2]. Such damage is usually irreversible and has a high probability of causing the patients’ body dysfunction and, in severe cases, death [3]. Statistics show that stroke inflicts approximately 800,000 people worldwide annually, and the incidence shows a gradual upward trend with the accelerated population aging [4,5]. Cerebral ischemic stroke (CIS) is the most common type of stroke, accounting for about 60–70% of all cases [6]. CIS, mainly induced by occlusion and stenosis of the internal carotid and vertebral arteries, is the most dangerous type of stroke [7]. At present, the clinical diagnosis and treatment for CIS are still complicated, which can only be determined by the relevant blood markers combined with head imaging technology, and the treatment is mainly surgery [8,9]. As such, most CIS patients cannot receive timely, rapid and effective treatment, resulting in irreversible neurological damage and even life threat [10]. Hence, finding a new CIS diagnosis and treatment scheme is a trending topic in clinical research.

With the development and deepening of medical research, accumulating researchers are focusing on the occurrence of diseases caused by genetic changes. Among them, microRNA (miRNA), as a representative, has been extensively studied in CIS [11,12]. miRs, as human genetic genes, play an important role in modulating heredity and changing cell biological behavior [13]. In related studies, we found that miR-497 is one of miRs that have been found to be vital in cerebrovascular diseases (such as cerebral ischemia-reperfusion injury and Parkinson’s disease) [14,15], and it has a strong regulatory effect on angiogenesis and neuroregulatory protein degradation [16,17]. But a link to CIS has not yet been confirmed. We hypothesized that miR-497 affected nerve cells through cerebral ischemia-reperfusion injury, which in turn affected the prognosis of patients with CIS. Accordingly, this study conducted an experimental analysis of the relationship between miR-497 and CIS, with the hope of providing a reliable basis for clinical response to CIS and laying a solid foundation for follow-up research.

## 2. Materials and methods

### 2.1. Patient information

This investigation enrolled 89 CIS patients and 39 physical examinees referred to our hospital from June 2017 to October 2018. Among the CIS patients, 22 cases had transient ischemic attack (TIA), 19 cases had reversible ischemic neurologic deficit (RIND), 28 cases had stroke in progression (SIP), and 20 cases had complete stroke (CS). CIS patients ranged in age from 48 to 70 years, with a mean age of (59.96 ± 7.24) years, and there were 46 males and 43 females. Thirty-nine physical examinees ranged in age from 48 to 70 years, with a mean age of (59.21 ± 7.18) years, and there were 20 males and 19 females. The internal Ethical Committee approved the study without reserves, and all the enrolled participants have signed the informed consent.

### 2.2. Inclusion and exclusion criteria

Patients diagnosed as CIS after examination in our hospital were included, who had complete case data and agreed to cooperate with medical staff in our hospital for investigation. Patients with complicated tumors, other cardiovascular and cerebrovascular diseases, other autoimmune deficiency diseases, infectious diseases, and mental diseases were excluded; Patients with organ dysfunction or drug allergy were excluded; Patients who had received antibiotics and surgery within 3 months prior to admission were excluded; Patients during pregnancy or lactation were excluded; Referred patients were excluded; Patients with a life expectancy of <1 month were excluded [18].

### 2.3. Treatment scheme

After admission, all CIS patients were treated according to 2019 American Heart Association (AHA)/American Stroke Association (ASA) International Stroke Conference (CIS) treatment guidelines [19]. After head and neck angiography, the scheme of intravenous thrombolysis or mechanical thrombectomy was decided, and follow-up antithrombotic and anticoagulant therapy was implemented.

### 2.4. Blood sampling

4 mL of fasting venous blood was drawn from physical examinees at admission and from CIS patients both at admission and discharge. After standing at room temperature for 30 min, serum was obtained by centrifugation (1505 × g, 4℃) and placed in a refrigerator at −80℃ for measurement.

### 2.5. Nerve defect score

The neurological function of CIS patients was evaluated by the National Institutes of Health Stroke Scale (NIHSS). The score range was 0–42 points, with 0–1 indicating normal/nearly normal, 1–4 indicating mild stroke, 5–15 indicating moderate stroke, 15–20 indicating moderate-severe stroke, and 21–42 indicating severe stroke [20].

### 2.6. miR-497 detection

Total RNA in serum was extracted by miRNA extraction and separation kit (Tiangen Biotech (Beijing), centrifugal column type, O4208) for reverse transcription, followed by PCR detection using a RealMasterMix Probe kit (Tiangen Biotech (Beijing), O4215). Reaction system and circulation conditions: 8 μL 2.5× RealMasterMix, 1 μL each of forward primer and reverse primer (5 pmol/L; designed by Thermo Fisher Scientific, see Table 1 for detailed sequences), 1 μL 20× Probe Enhancer solution, 1 μL template and 7 μL ddH2O; 94℃ for 2 min; 94℃ for 20 s; 60℃ for 30 s; 68℃ for 1 min; for 43 cycles. The expression level of miR-497 was analyzed by 2^−ΔΔCt^ with U6 as internal reference [21].

**Table 1.** Primer sequence

	Forward primer (5ʹ-3ʹ)	Reverse primer (5ʹ-3ʹ)
miR-497	ACCCAGAAGACTGTGGATGG	TTCCTTCAGAGCAAACAG-CA
U6	AGGGGAGATTCAGTGTGGTG	GTTGTGCTCAA ATCCCCATT

### 2.7. Detection of oxidative stress response of patients

Measurements of malondialdehyde (MDA) and superoxide dismutase (SOD) were performed using Enzyme-linked immunosorbent assay (ELISA) kits specific for MDA (Shanghai Lengton Biotech BP-E10376) and SOD (Shanghai Xinfan Biotech, XFE1339A). The operation process was in strict conformity with the kit instructions.

### 2.8. Follow-up of prognosis

All CIS patients were followed up for a 3-year hospital review to record the disease recurrence rate and mortality during this period.

### 2.9. Animal information

Thirty healthy adult male SD rats, weighing 250–280 g and aged 10–14 weeks, were acquired from Beijing Novo Nordisk Pharmaceutical Technology, with the license number of SCXK (Beijing) 2018–0017. Ten rats were targeted to increase miR-497 expression, and the other 20 rats were normally fed.

Overexpression vector miR-497 mimics was purchased from Shanghai Wansheng Haotian Biotechnology Co., Ltd. MiR-497 overexpressing rats were constructed according to the following steps. (1) 0.67 μg (50 pmol) miR-497 mimics was added to a certain amount of serum-free diluent and mixed thoroughly to prepare RNA Diluent. (2) Mix EntransterTM-R4000 diluent and RNA diluent thoroughly. The preparation of the transfection complex is complete. (4) 50 μl of transfection complex was added dropwise to the cells with 0.45 ml of complete medium. (5) Observe the cell status 6 h after transfection. Finally, the transfected cells were injected into mice.

### 2.10. Animal grouping and modeling

The 10 rats targeted to increase miR-497 expression were allocated into group A, and the other 20 rats were randomized into groups B (n = 10) and C (n = 10). Middle cerebral artery occlusion (MACO) was modeled in groups A and B [22]. After anesthesia with 3% Pentobarbital sodium (30 mg/kg), the rats were fixed in supine position, and the right common carotid artery (CCA), internal carotid artery (ICA) and external carotid artery (ECA), as well as the branches of the right ECA and the right ICA were exposed by incision in the middle of the neck, with seton placement. Then, the ECA branch was ligated and cut short, and the right CCA was temporarily blocked with artery clamps. The ECA was ligated with double silk threads 0.8–1.0 cm away from the bifurcation of the right CCA. Thereafter, a small incision was made at the proximal end of the right ECA, and a smooth spherical poly-lysine treated 4–0 nylon thread (0.25 mm in diameter) was inserted at one end and gently inserted into ICA at the bifurcation. When the nylon thread diverged from the CCA by 1.8–2.0 cm and slight resistance was felt, the proximal end of the ECA where the nylon thread was inserted was ligated with silk thread. In sham operation group, nylon thread was inserted only 0.3–0.6 cm without blocking the middle cerebral artery. After 60 min of ischemia, the nylon thread was drawn out and the arterial clamp of the CCA was loosened for perfusion. This study was approved by the Institutional Animal Care and Use Committee.

### 2.11. Rat brain tissue weighing

Rat brain moisture content in was measured by wet-dry weighting method [23]: all rats were killed under anesthesia 24 h after the completion of modeling, and the brain tissue was obtained. The brainstem, cerebellum and right cerebral hemisphere were removed, and the remaining left cerebral hemisphere was weighed immediately to obtain wet weight (WW). Then, the cerebral hemisphere was dried at 72℃ for 72 h to measure dry weight (DW). Brain water content was calculated as (WW-DW)/WW×100%.

### 2.12. Neuronal apoptosis in brain tissue

TUNEL assay was utilized for apoptosis detection in rat brain tissue [24]: all brain samples were sliced (6 *μ*m) and cultivated in darkness with TUNEL reaction mixture for 2 h at ambient temperature. Then, the slices were washed with PBS and incubated with DAPI for 5 min. After all slides were rinsed three times in PBS, the area around the damaged center was examined by covering the cover glass with an anti-fluorine quenching sealant under the microscope. The final mean value of apoptotic index calculated from four sections was used as data for each sample.

### 2.13. Statistical processing

Data analysis and processing were made by SPSS22.0 statistical software. The counting data was calculated as percentage, recorded as (%), and compared using the chi-square test. For measurement data, the mean and standard deviation were calculated and recorded as (mean ± standard deviation), and were compared by independent sample T-test, paired T-test, one-way ANOVA and LSD post-hoc test. Pearson correlation coefficient was utilized for correlation analyses, and the predicted significance of miR was analyzed by the ROC curve. When *P* values <0.05, statistically significant differences were indicated [25].

## 3. Results

**Figure 1.**
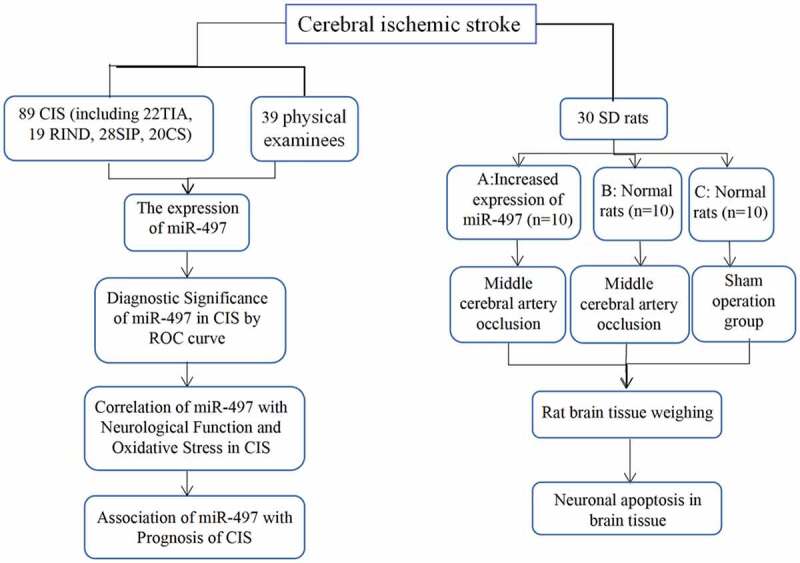
The flow table for this study

We hypothesize that miR-497 affects nerve cells through cerebral ischemia-reperfusion injury, which in turn affects the prognosis of patients with CIS. But a link to CIS has not yet been confirmed. Accordingly, this study conducted an experimental analysis of the relationship between miR-497 and CIS, with the hope of providing a reliable basis for clinical response to CIS and laying a solid foundation for follow-up research. The flow table for this article is shown in Figure 1.

### 3.1. miR-497 expression in CIS

**Figure 2.**
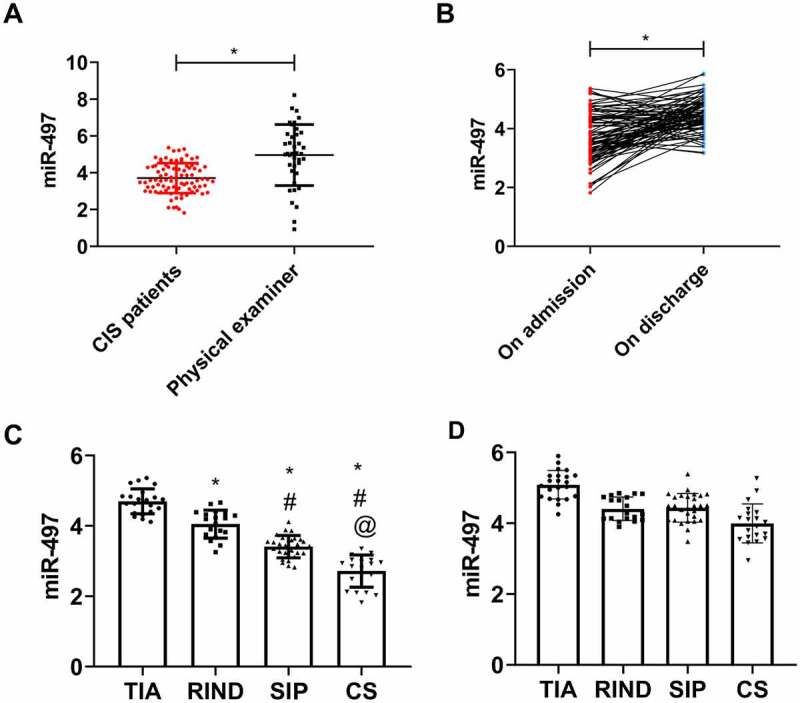
miR-497 expression in CIS. (a) Serum miR-497 expression in CIS patients and health examinees on admission; (b) miR-497 expression in CIS patients on admission and discharge; * *P* < 0.05. (c) miR-497 expression in four types of CIS patients on admission. * *P* < 0.05 *vs*. TIA; # *P* < 0.05 *vs*. RIND; @ *P* < 0.05 *vs*. SIP. (d) miR-497 expression in four types of CIS patients on discharge

On admission, serum miR-497 was lower in CIS patients (3.71 ± 0.81) than in physical examinees (4.96 ± 1.66) (*P* < 0.05, Figure 2(a)). On discharge, however, miR-497 in CIS patients was 4.49 ± 0.56, which was distinctly higher than that on admission (*P* < 0.05, Figure 2(b)). Among the four types of CIS, miR-497 was the lowest in CS patients, followed by SIP and RIND patients, with that in TIA patients being the highest (*P* < 0.05, Figure 2(c)).

### 3.2. Diagnostic significance of miR-497 in CIS

**Figure 3.**
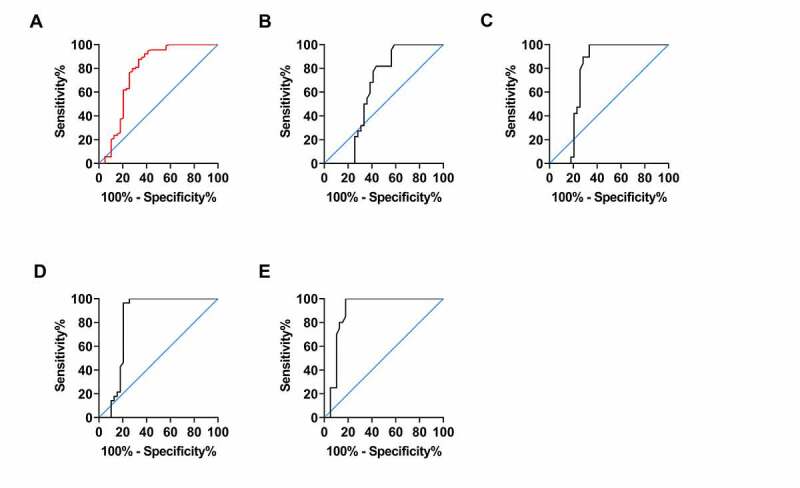
Diagnostic significance of miR-497 in CIS. (a) ROC curve of CIS occurrence predicted by miR-497. (b) ROC curve of TIA occurrence predicted by miR-497. (c) ROC curve of RIND occurrence predicted by miR-497. (d) ROC curve of CS occurrence predicted by miR-497. (e) ROC curve of SIE occurrence predicted by miR-497

ROC curve analysis using miR-497 expression in CIS patients and healthy examinees showed that when miR-497 < 4.690, the sensitivity and specificity in predicting CIS occurrence were 87.64% and 66.67%, respectively (*P* < 0.001, Figure 3(a)). However, no distinct difference was observed in miR-497 expression between TIA patients and physical examinees, making ROC analysis inapplicable (*P* > 0.05, Figure 3(b)). ROC analysis using miR-497 in RIND patients and physical examinees exhibited that when miR-497 < 4.685, the sensitivity in predicting RIND was 100.0%, and the specificity was 66.67% (*P* < 0.05, Figure 3(c)). ROC analysis of miR-497 expression from SIE patients and physical examinees revealed that the sensitivity and specificity of miR-497 < 3.925 in predicting SIE were 96.43% and 79.49%, respectively (*P* < 0.001, Figure 3(d)). While ROC analysis using miR-497 expression in CS patients and physical examinees showed that the sensitivity in predicting CS was 100.0%, and the specificity was 82.05, when miR-497 < 3.385 (*P* < 0.001, Figure 3(e)). The relevant data of ROC curve analyses are presented in Table 2.

**Table 2.** Diagnostic significance of miR-497 in CIS by ROC analysis

	CIS	TIA	RIND	SIP	CS
AUC	0.7748	0.6265	0.7578	0.8182	0.8936
Std.Error	0.0554	0.07135	0.06519	0.0582	0.0446
95%CI	0.6662–0.8835	0.4866–0.7663	0.6300–0.8855	0.7041–0.9323	0.8065–0.9807
P	<0.001	0.1032	0.0016	<0.001	<0.001
Cutoff	<4.690	-	<4.685	<3.925	<3.385
Sensitivity (%)	87.64	-	100.0	96.43	100.0
Specificity (%)	66.67	-	66.67	79.49	82.05

### 3.3. Correlation of miR-497 with neurological function and oxidative stress in CIS patients

**Figure 4.**
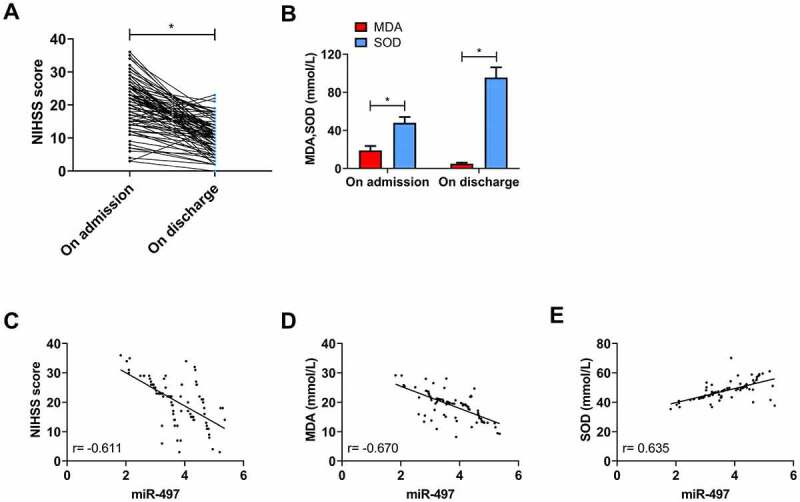
Correlation of miR-497 with neurological function and oxidative stress in CIS patients. (a) NIHSS scores of CIS patients on admission and discharge; (b) Serum MDA and SOD concentrations of CIS patients on admission and discharge; * *P* < 0.05. (c) Correlation analysis between miR-497 and NIHSS score in CIS patients on admission; (d) Correlation analysis between miR-497 and MDA concentration in CIS patients on admission; (e) Correlation analysis between miR-497 and SOD concentration in CIS patients on admission

The NIHSS score of patients was (20.39 ± 7.43) on admission and significantly decreased on discharge (*P* < 0.05, Figure 4(a)). On admission, MDA and SOD were (19.01 ± 4.60) mmol/L and (48.04 ± 6.15) mmol/L, respectively; however, MDA decreased and SOD increased on discharge (*P* < 0.05, Figure 4(b)). An inverse association was determined between miR-497 and NIHSS score and MDA concentration on admission by Pearson correlation coefficient analysis (r = −0.611, −0.670; *P* < 0.001, Figure 4(c,d)), while a positive correlation between miR-497 and SOD concentration (r = 0.635; *P* < 0.001, Figure 4(e)).

### 3.4. Association of miR-497 with prognosis of CIS patients

**Figure 5.**
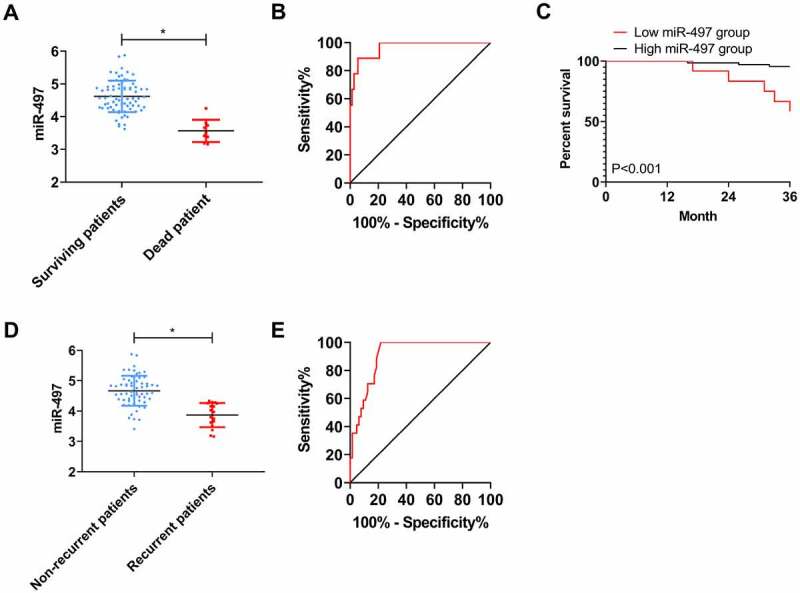
Association between miR-497 and prognosis of CIS patients. (a) miR-497 levels in patients with prognostic death and survival on discharge; * *P* < 0.05. (b) ROC curve of 3-year death predicted by miR-497 on discharge; (c) 3-year survival curves of patients in high miR-497 group and low miR-497 group; (d) miR-497 levels in patients with and without recurrence on discharge; * *P* < 0.05. (e) ROC curve of 3-year recurrence predicted by miR-497 on discharge

Eighty-one patients were successfully followed up during the 3-year follow-up, with a follow-up success rate of 91.01%. The overall 3-year mortality rate of the patients was 9.88% (8/81). Comparing the level of miR-497 between the dead and the survival on discharge, it can be seen that miR-497 was notably lower in the dead patients than in the surviving patients (*P* < 0.05, Figure 5(a)). ROC analysis using miR-497 expression in dead patients and surviving patients on discharge exhibited that when miR-497 < 3.840 on discharge, the sensitivity and specificity of predicting 3-year death of patients were 88.89% and 94.44%, respectively (*P* < 0.001, Figure 5(b)). Further, patients were assigned to high- (miR-497 ≥ 3.840) or low- (miR-497 < 3.840) group of miR-497 according to the cutoff value. Observing the prognostic survival curves, we can find distinctly higher prognostic mortality in the low miR-497 group as compared to high miR-497 group (*P* < 0.001, Figure 5(c)). The recurrence rate of CIS patients within 3 years was 20.99% (17/81). Comparison of miR-497 in patients with and without recurrence on discharge revealed evidently lower miR-497 levels in patients with recurrence than in those without (*P* < 0.05, Figure 5(d)). ROC analysis using miR-497 in patients with recurrence and those without on discharge revealed that when miR-497 < 4.335 on discharge, the sensitivity and specificity for predicting 3-year recurrence were 100.0% and 78.13%, respectively (*P* < 0.001, Figure 5(e)).

### 3.5. Impact of elevated miR-497 on CIS rats

**Figure 6.**
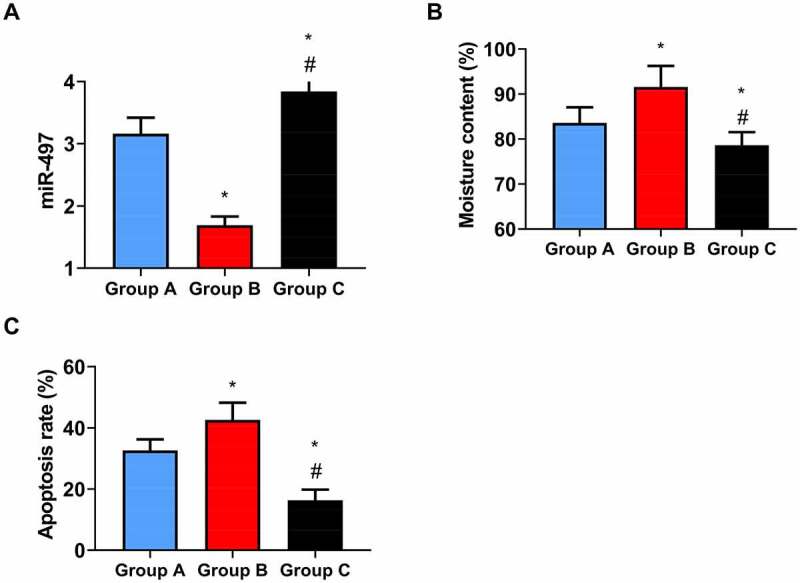
Impact of elevated miR-497 on CIS rats. (a) miR-497 levels in rat brain tissue in the three series; (b) Water contents of rat brain tissue in the three series; (c) Neuronal apoptosis in rat brain tissue in the three series; * *P* < 0.05 *vs*. group A, # *P* < 0.05 *vs*. group B

First of all, miR-497 was found to be highest in group C, and was higher in group A as compared to group B (*P* < 0.05, Figure 6(a)). The water content of rat brain tissue was the lowest in group C, and was lower in group A as compared to group B (*P* < 0.05, Figure 6(b)). The detection of neuronal apoptosis in rat brain tissue in the three series showed that the neuronal apoptosis rate in group C was the lowest, followed by group A, and the apoptosis rate in group B was the highest (*P* < 0.05, Figure 6(c)).

## 4. Discussion

CIS, as a critical illness with a high incidence in the world, poses a great potential threat [26]. Due to the complexity of its diagnosis and treatment scheme, the potential risk of CIS is further increased [27]. So, a deep understanding of the molecular pathogenesis of CIS is a hot and difficult point in clinical research [28]. This study, by exploring the correlation of miR-497 with CIS, has the following important clinical implications. 1. As genetic genes in human body, miRs can be detected in blood, body fluids, tissues, cells and other substances with relatively convenient and fast methods. At present, the diagnosis of CIS is extremely complicated, which requires not only a great deal of time and energy, but also a high economic expenditure. Moreover, the recurrence diagnosis scheme cannot realize the large-scale clinical screening, nor to improve the early diagnosis rate of CIS or reduce the threat posed by CIS. If the diagnostic significance of miR-497 in CIS could be confirmed, the current diagnostic status can be greatly improved by using miR-497 as a marker of CIS in the future. 2. For the determination of CIS progression, it is also necessary to rely on a number of examination methods, whereas the most important evaluation index, imaging technology, is mainly based on the subjective judgment of the film reader, with low objectivity, and it is even more difficult to judge the CIS with rare lesions [29]. After understanding the correlation of miR-497 with the disease progression of CIS, the condition of patients can be objectively evaluated by observing the changes of miR-497, allowing for timely and effective intervention to reduce the risk of CIS. 3. The key treatment for CIS is thrombolysis and thrombectomy, but mechanical invasive surgery may increase oxidative stress injury of the patient’s brain tissue and cause irreversible nerve damage [30]. Moreover, brain surgery, as the most difficult class in clinical practice, has extremely strict requirements for doctors. Therefore, if molecular targeted therapy can be adopted in the future, it will not only greatly cut down the treatment cost, but also achieve better treatment effect than the current clinical surgery due to the treatment of CIS from the perspective of genetic changes. Accordingly, we launched a preliminary analysis of miR-497 and CIS taking into the above points into consideration.

First, we detected miR-497 expression in peripheral blood of CIS patients and healthy examinees, and determined lower miR-497 levels in patients, suggesting that miR-497 was abnormally expressed in CIS, which may be involved in the occurrence and development of CIS. In previous studies, we also found that miR-497 was low expressed in diseases like liver cancer and skin cancer [31,32], which can also testify our experimental results. Then, we observed miR-497 levels in patients on discharge and found evidently increased levels after treatment, further suggesting that miR-497 is strongly related to the changes of CIS, and that elevated miR-497 may alleviate CIS. Besides, it was observed that among four different types of CIS, miR-497 was the lowest in patients with CS, followed by SIP and RIND, and miR-497 expression in TIA patients was the highest. Referring to the severity of four CIS types [33], we can confirm once again that the decrease of miR-497 predicts the severity of CIS progression.

Then, we analyzed the clinical implications of miR-497 in CIS. First, through ROC curve analysis, we found that when miR-497 < 4.690, the sensitivity and specificity to predict the occurrence of CIS were 87.64% and 66.67%, respectively, and the sensitivity gradually increased with the severity of the patient’s disease; hence, we can preliminarily confirm that miR-497 has the potential to be a CIS marker. However, due to the small number of cases included in this study, the optimal cutoff value of miR-497 for CIS prediction still needs to be analyzed by expanding the sample size. Pearson correlation coefficient analysis showed that miR-497 was negatively correlated with NIHSS score and MDA concentration, and positively related to SOD concentration in CIS patients at admission. The mechanism of miR-497 inducing oxidative stress response may be cell injury induced by ischemia/reperfusion injury [14]. The NIHSS is one of the crucial observation indexes for the evaluation of nerve injury in cerebrovascular diseases; the higher the NIHSS score, the more serious the nerve injury of patients [34]. While MDA and SOD are classic markers of oxidative stress reaction. MDA is one of the essential products of membrane lipid peroxidation, and its elevated level can aggravate the damage of cell and tissue membrane [35]. SOD, as an antioxidant metallase, can catalyze superoxide anion radical disproportionation to produce oxygen and hydrogen peroxide [36]. A number of studies have shown that one of the ways that CIS damages brain tissue cells is tissue cell necrosis caused by oxidative stress injury [37,38], which can also be confirmed by observing the concentration of MDA and SOD at admission and discharge, so we will not repeat it here. The correlation of miR-497 expression with NIHSS score, MDA, and SOD identified in the present study can further confirm that miR-497 interferes with CIS progression, suggesting that monitoring miR-497 levels in CIS patients can help clinicians to judge patients’ condition in the future. Once again, in the prognostic follow-up, we found that the miR-497 level decreased statistically in patients with prognostic death and disease recurrence on discharge, and ROC analysis based on miR-497 expression in patients at discharge was found to be highly effective in predicting the prognosis of death and relapse of patients, demonstrating the great potential of miR-497 as a marker of CIS. Moreover, the observation of prognosis survival curves of CIS patients with high or low miR-497 expression further indicated that decreased miR-497 after treatment is indicative of the poor prognosis of patients.

Finally, in order to understand the specific mechanism of miR-497 on CIS, we observed the water content of brain tissue and the neuronal apoptosis of the three series of rats by establishing a CIS rat model. The results showed that compared with normal CIS rats, the water content of brain tissue and the neuronal apoptosis rate in CIS rats with targeted elevation of miR-497 were statistically lower. During the occurrence of CIS, neuronal cell injury and apoptosis, edema caused by increased permeability of blood–brain barrier and increased water content are all extremely obvious pathological reactions [39,40]. And our experimental results indicate that increasing miR-497 can effectively reduce the brain injury of CIS rats, providing a basis for future targeted therapy through miR-497. Related studies have also shown that serum miR-497 levels can be used as a biomarker for the prognosis of patients with acute cerebral infarction [41]. In addition, inhibiting miR-497 can improve the functional outcome after ischemic stroke by enhancing neuronal autophagy in rats [42].

However, there are still some limitations to be addressed. First, as aforementioned, the number of cases included in the study is too small to obtain the most accurate and representative results. Second, we have not analyzed the exact influencing mechanism of miR-497 on CIS. Moreover, the short experimental period resulted in the inability to evaluate the relationship between miR-497 and the long-term prognosis of CIS patients. We will conduct further experimental investigation to address the above shortcomings as soon as possible to obtain more comprehensive and complete experimental results for clinical reference.

## 5. Conclusion

Reduced miR-497 levels may indicate severe disease progression and poor prognosis of CIS patients. miR-497 is of great significance in CIS diagnosis and treatment and has the potential to be a CIS marker. The mechanism of miR-497 on CIS may be through intensifying oxidative stress and promoting neuronal apoptosis.

## Highlights

1. miR-497 expression is lower in Cerebral Ischemic Stroke (CIS) patients.
2. miR-497 is closely associated with Poor Prognosis in Patients with CIS.
3. Overexpression of miR-497 inhibits brain injury in CIS rats.
